# Study of Cyclic Quaternary Ammonium Bromides by B3LYP Calculations, NMR and FTIR Spectroscopies

**DOI:** 10.3390/molecules15085644

**Published:** 2010-08-16

**Authors:** Bogumil Brycki, Adrianna Szulc, Iwona Kowalczyk

**Affiliations:** Laboratory of Microbiocides Chemistry, Faculty of Chemistry, A. Mickiewicz University, Grunwaldzka 6, 60-780 Poznań, Poland; E-Mails: iwkow@amu.edu.pl (I.K.); adrszu@gmail.com (A.S.)

**Keywords:** *N,N*-dioctyl-azepanium, -piperidinium, -pyrrolidinium bromides, DFT calculations, FTIR and NMR spectra

## Abstract

*N,N*-dioctyl-azepanium, -piperidinium and -pyrrolidinium bromides **1**-**3**, have been obtained and characterized by FTIR and NMR spectroscopy. DFT calculations have also been carried out. The optimized bond lengths, bond angles and torsion angles calculated by B3LYP/6-31G(d,p) approach have been presented. Both FTIR and Raman spectra of **1-3** are consistent with the calculated structures in the gas phase. The screening constants for ^13^C and ^1^H atoms have been calculated by the GIAO/B3LYP/6-31G(d,p) approach and analyzed. Linear correlations between the experimental ^1^H and ^13^C chemical shifts and the computed screening constants confirm the optimized geometry.

## 1. Introduction

Quaternary ammonium compounds (QACs) were introduced as antimicrobial agents by Domagk over seventy years ago [1]. The first generation of QACs were standard benzalkonium chlorides, *i.e. *alkylbenzyldimethylammonium chloride, with specific alkyl distributions, *i.e., *C_12_, 40%; C_14_, 50% and C_16_, 10% [2]. The second generation of QACs was obtained by substitution of the aromatic ring in alkylbenzyldimethylammonium chlorides by chlorine or alkyl groups to get products like alkyldimethylethylbenzylammonium chloride with C_12_, 50%; C_14_, 30%; C_16_, 17% and C_18_, 3% alkyl distribution. Dual quaternary ammonium salts are the third generation of QACs. These products are a mixture of equal proportions of alkyldimethylbenzylammonium chloride with alkyl distribution C_12_, 68%; C_14_, 32% and alkyldimethylethylbenzylammonium chloride with alkyl distribution C_12_, 50%; C_14_, 30%; C_16_, 17% and C_18_, 3%. The twin chain quaternary ammonium salts, like didecyldimethyl-ammonium chloride are the fourth generation of QACs. The concept of synergistic combinations of dual QACs has been applied to twin chain quaternary ammonium salts. The mixture of dialkyldimethylamoonium chloride (dioctyl, 25%; didecyl, 25%, octyldecyl, 50%) with benzalkonium chloride (C_12_, 40%; C_14_, 50%; C_16_, 10%) is the newest blend of quaternary ammonium salts which represents the fifth generation of QACs [2]. Because of the increasing resistance of microorganisms to commonly used disinfectants, the synthesis of new types of microbiocides is very important. One of the new groups with good antimicrobial activity are the cyclic quaternary ammonium salts [3]. The aim of this work was the synthesis of cyclic *N,N*-dioctyl quaternary ammonium salts, *i.e. N,N*-dioctyl-azepanium, *N,N*-dioctylpiperidinium and *N,N*-dioctylpyrrolidinium bromides, with potential antimicrobial activity. Some cyclic quaternary ammonium salts have previously been obtained by intramolecular cyclisation of amine derivatives [4,5,6,7,8,9]. Another way, *i.e. *reaction of alkyl halides with cyclic amines, can lead to chiral cyclic quaternary ammonium salts [10].

In recent years numbers of applications of the quaternary ammonium salts has been continuously increasing. They are used as biocides [11,12,13,14,15], and phase-transfer catalysts, especially in enantioselective reactions [16,17,18,19,20,21]. Pyrrolidinium salts are analogues of oxotremorine and are used as muscarinic agonists [5]. Some quaternary ammonium salts exist as ionic liquids, which can be used as “green solvents” [22,23,24,25,26] and electrolytes for liquid batteries [27,28].

The molecular structures of *N,N*-dioctyl-azepanium (**1**), -piperidinium (**2**) and -pyrrolidinium (**3**) bromides analyzed by FTIR and NMR spectroscopy and B3LYP calculations are presented in this paper. The above compounds belong to the cyclic quaternary ammonium bromide family investigated in our laboratory in order to better understand the mechanism of their biological activity.

## 2. Results and Discusion

### 2.1. Synthesis

*N,N-*dioctyl-azepanium, -piperidinium and -pyrrolidinium bromides **1-3** were obtained by reaction of *N,N*-dioctylamine with dibromohexane, dibromopentane and dibromobutane, respectively. The reaction of secondary amines with 1,5-dichloropentane and 1,4-dichlorobutane to produce dialkylpiperidinium and dialkylpyrrolidinium salts has previously been described by Ericsson and Keps [4]. In our work, using dibromoalkanes instead of dichloroalkanes, we formed five-, six- and seven-membered ammonium compounds in much higher yields and after shorter reaction times. In the first step of reaction of dioctylamine with α,ω-dibromoalkane, the halogenated tertiary amine is formed, which shows a strong tendency to form cyclic quaternary ammonium salts.

### 2.2. B3LYP Calculations

**Figure 1:**
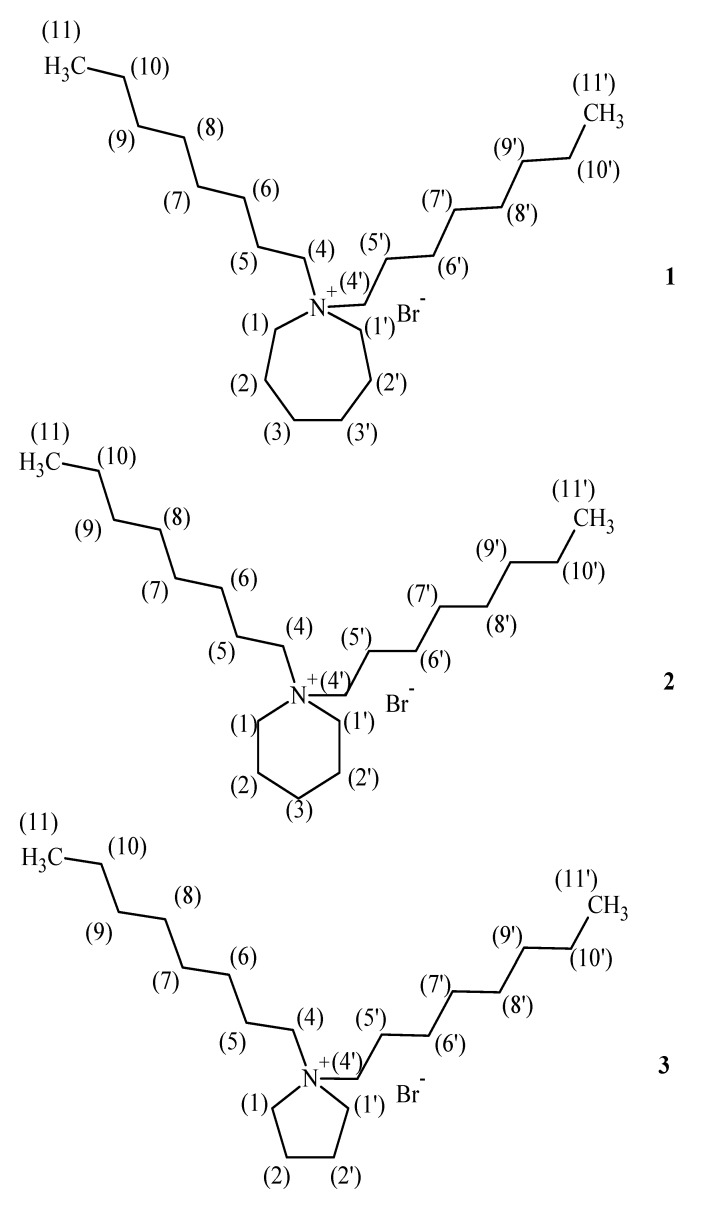
The structure and numbering for *N,N*-dioctylazepaniumbromide (**1**), *N,N*-dioctyl-piperidinium bromide (**2**) and *N,N*-dioctylpyrrolidinium bromide (**3**).

**Figure 2:**
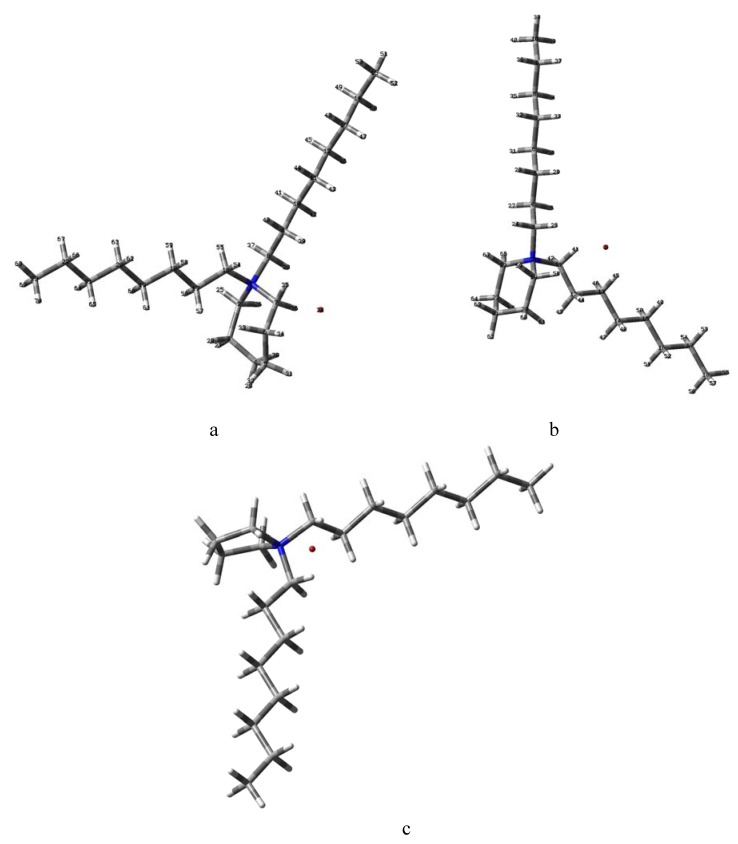
Structures of (a) *N,N*-dioctylazepanium (**1**), (b) *N,N*-dioctylpiperidinium (**2**), (c) *N,N-*diocylpyrrolidinium (**3**) bromides optimized by the B3LYP/6-31G(d,p) method.

The structures and numbering for **1-3** are given in Figure 1. The structures optimized at the B3LYP/6-31G(d,p) level of theory are shown in Figure 2. 

The computed B3LYP geometry parameters, energy and dipole moments are given in Table 1. The calculated energy for *N,N*-dioctylazepanium bromide (**1**) is about 1.2% lower than for *N,N*-dioctyl-piperidinium bromide (**2**) and 2.4% lower in comparison to *N,N*-dioctylpyrrolidinium bromide (**3**). The bromide anions in **1-3** are engaged in three non-linear weak intramolecular interactions with carbon atoms. Bromide anions additionally interact *via* Coulombic attractions with positively charged nitrogen atom. The N^+^(…)···Br^-^ distances are 3.888 Å, 3.709 Å 3.674 Å, for **1**, **2** and **3**, respectively.

**Table 1:** Selected parameters of investigated molecules **1-3** estimated by B3LYP/6-31G(d,p) calculations.

Parameters	1	2	3
Energy (a.u)	-3495.20808	-3453.27811	-3413.96044
Dipol moment (Debye)	13.4951	11.4097	11.4657
*Bond length (Å)*			
N^+^…Br^-^	3.888	3.709	3.674
C(1)-H…Br^-^	3.636	3.536	3.486
C(1’)-H…Br^-^	3.686		
C(4)-H…Br^-^	3.551	3.570	3.616
C(4’)-H…Br^-^		3.360	3.346
N-C(1)	1.535	1.538	1.532
N-C(1’)	1.533	1.514	1.513
N-C(4)	1.548	1.542	1.529
N-C(4’)	1.531	1.551	1.543
*Bond angle (^o^)*			
N-C(1)-C(2)	119.5	115.3	106.2
N-C(1’)-C(2’)	116.9	114.2	106.2
N-C(4)-C(5)	117.9	116.3	115.6
N-C(4’)-C(5’)	120.2	119.9	118.6
*Dihedral angle (^o^)*			
N-C(1)-C(2)-C(3)	-70.3	-49.5	
N-C(1’)-C(2’)-C(3’)	88.6		
N-C(1’)-C(2’)-C(3)		57.8	
N-C(1)-C(2)-C(2’)			-18.2
N-C(1’)-C(2’)-C(2)			25.2
N-C(4)-C(5)-C(6)	-176.8	-177.4	-176.9
N-C(4’)-C(5’)-C(6’)	-176.5	-172.3	-170.0

### 2.3. FTIR and Raman Spectra Study

**Figure 3:**
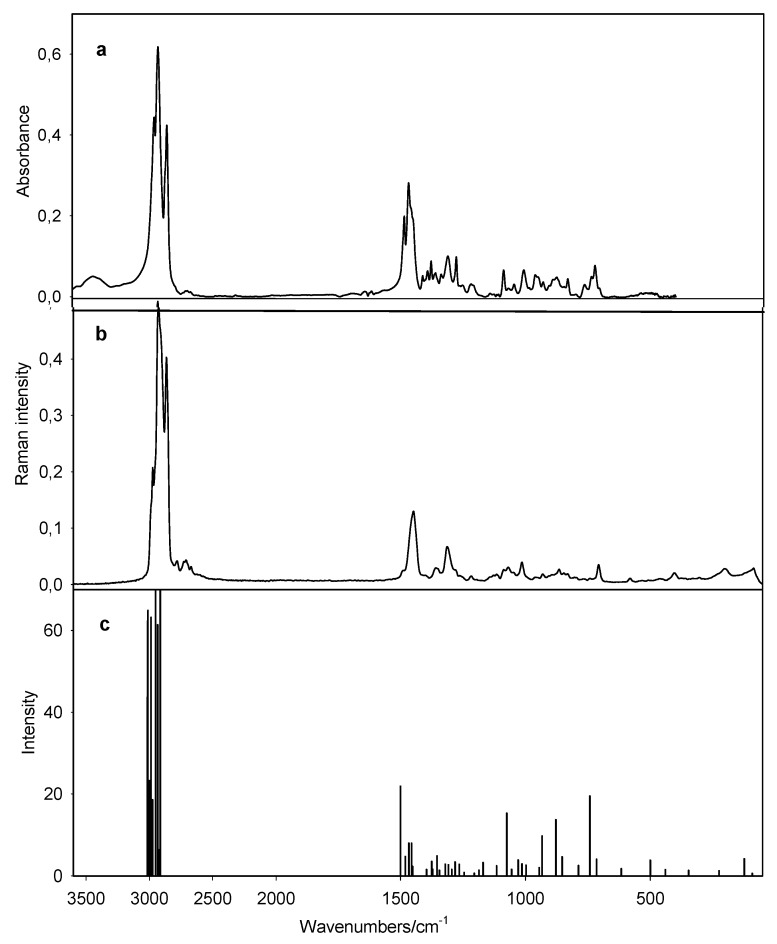
Spectra of *N,N*-dioctylazepanium bromide (**1**);(a) FTIR, (b) Raman and (c) calculated spectra.

Room-temperature solid-state FTIR and Raman spectra as well as the calculated spectra of **1** are shown in Figure 3. 

The observed and calculated harmonic frequencies and their tentative assignments are listed in Table 2. In general, the calculated frequency values with B3LYP 6-31G(d,p) basis set are close to experimental values of vibrational frequency.

**Table 2:** FTIR and Raman frequencies of *N,N*-dioctylazepanium bromide (**1**).

Raman	IR	IR(calc.)	INT	Proposed assignment
	3437w			νOH
2973m	2956s	3016	43.7	νCH_2_
		3013	62.3	νCH_2_
		3011	64.9	νCH_2_
		2999	23.3	νCH_2_
		2987	63.2	νCH_2_
		2974	18.6	νCH_2_
		2943	112	νCH_2_
2926s	2925s	2934	61.4	νCH_2_
		2919	6.4	νCH_2_
2864s	2856s	2914	200	νCH_2_
2781vw				νCH_2_
2727vw				νCH_2_
2709vw	2696vw			νCH_2_
2669vw	2670vw			νCH_2_
1490vw	1485m	1501	21.9	νCC
		1481	4.7	
1448w	1468m	1467	8.0	νCC
		1456	7.9	
		1452	2.3	
	1392w	1396	1.5	νCN
	1377w	1376	3.5	νCN
		1372	1.6	
1358vw	1360w	1354	4.9	νCC, βCH_2_
1349vw	1338w	1344	1.4	βCH_2_
		1321	2.8	
1313vw	1310w	1308	2.7	βCH_2_
		1295	1.6	
1280vw	1277w	1281	3.4	νCC
1263vw	1251vw	1264	2.8	νCC
		1245	0,81	
1217vw	1218vw	1205	0.63	νCC
		1186	1.3	
1141vw	1141vw	1169	3.3	νCN
1115vw	1115vw	1115	2.4	νCN
1087vw	1088w	1075	15.3	γCH_2_
1069vw	1068vw	1055	1.6	γCH_2_
1048vw	1047vw	1029	3.8	βCH_2_
1014vw	1007w	1014	2.9	βCCC
960vw	962w	997	2.6	βCCC
930vw	930vw	944	2.0	βCCC
		933	9.7	
865vw	875w	878	13.7	βCCC
846vw	847vw	853	4.6	βCCC
831vw	832w			βCCC
803vw	800vw	788	2.5	βCCC
767vw	765vw	742	19.5	βCCC
741vw	738w			βCCC
	723w	714	4.0	βCCC
706vw				βCCC
659vw	651vw	616	1.8	βCNC
580vw	578vw			βNCC
542vw	538vw			βCCC
498vw	499vw	499	3.8	γCCC
403vw	403vw	439	1.5	γCCC
375vw		346	1.3	Lattice mode
360vw				Lattice mode
330vw				Lattice mode
303vw				Lattice mode
288vw		224	1.2	Lattice mode
201vw		123	4.1	Lattice mode
86vw		91	0.59	Lattice mode
		51	2.5	

The abbreviations used are: s, strong; m, medium; w, weak; vw, very weak; ν, stretching; β, in plane bending; δ, deformation; γ, out of plane bending; and τ, twisting.

### 2.4. 1H-NMR and 13C-NMR Spectra

**Figure 4:**
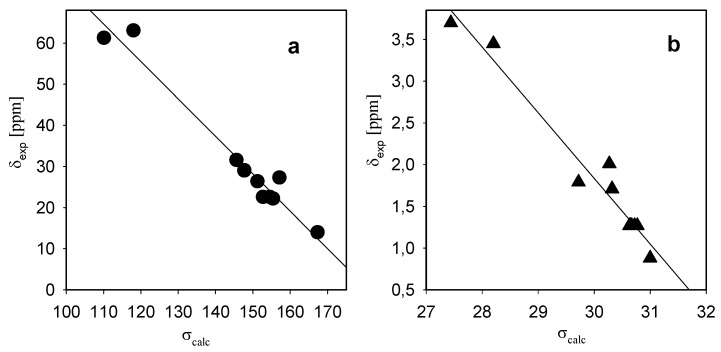
Plots of the experimental chemical shifts (δ_exp_) vs the magnetic isotropic shielding (σ_calc_) from the GIAO/B3LYP/6-31G(d,p); *N,N*-dioctylazepanium bromide (**1**) δ_pred_ = a + b· σ_calc_. (a) carbon-13; (b) proton.

The proton chemical shift assignments (Table 3-Table 5) are based on 2D COSY experiments, in which the proton-proton connectivity is observed through the off-diagonal peaks in the counter plot. The relations between the experimental ^1^H and ^13^C chemical shifts (δ_exp_) and the GIAO (Gauge-Independent Atomic Orbitals) isotropic magnetic shielding (σ_calc_) for **1** is shown in Figure 4. Both correlations are linear, described by the relationship: δ_exp _= a + b·σ_calc_. The parameters a and b are given in Table 3-Table 5. The very good correlation coefficients (r^2^=0.9379) for ^1^H and (r^2^=0.9984) for ^13^C confirm the optimized geometry of **1**-**3**.

**Table 3:** Chemical shifts (δ, ppm) in CDCl_3_ and calculated GIAO nuclear magnetic shielding (σ_cal_) for *N,N*-dioctylazepanium bromide (**1**). The predicted GIAO chemical shifts were computed from the linear equation δ_exp_= a + b·σ_calc_ with a and b determined from the fit the experimental data.

	δ_ exp_	δ_calc_	σ_calc_		δ_exp_	δ_calc_	σ_calc_
C(1)	63.1	57.4	118.0	H(1)	3.70	3.85	27.44
C(2)	22.2	23.3	155.4	H(2)	2.01	1.62	30.27
C(3)	27.3	21.7	157.1	H(3)	1.79	2.06	29.72
C(4)	61.3	64.6	110.1	H(4)	3.45	3.25	28.20
C(5)	22.6	25.7	152.7	H(5)	1.71	1.59	30.32
C(6)	26.4	27.1	151.2	H(6)	1.27	1.23	30.77
C(7)	29.1	30.3	147.7	H(7)	1.27	1.33	30.65
C(8)	29.0	30.3	147.7	H(8)	1.27	1.31	30.67
C(9)	31.6	32.2	145.6	H(9)	1.27	1.27	30.72
C(10)	22.6	24.1	154.5	H(10)	1.27	1.34	30.63
C(11)	14.0	12.4	167.3	H(11)	0.88	1.05	31.00
a		-0.9113		a		-0.7865	
b		164.9046		b		25.4318	
r^2^		0.9622		r^2^		0.9609	

**Table 4:** Chemical shifts (δ, ppm) in CDCl_3_ and calculated GIAO nuclear magnetic shielding (σ_cal_) for *N,N*-dioctylpiperidinium bromide (**2**). The predicted GIAO chemical shifts were computed from the linear equation δ_exp_= a + b·σ_calc_ with a and b determined from the fit the experimental data.

	δ_ exp_	δ_calc_	σ_calc_		δ_exp_	δ_calc_	σ_calc_
C(1)	58.9	54.8	129.6	H(1)	3.78	3.28	27.90
C(2)	20.0	20.5	167.8	H(2)	1.90	1.67	29.95
C(3)	26.4	20.5	167.8	H(3)	1.90	1.47	30.23
C(4)	58.1	61.0	122.7	H(4)	3.46	3.88	27.12
C(5)	21.7	23.2	161.8	H(5)	1.65	1.77	29.84
C(6)	22.5	25.7	162.1	H(6)	1.27	1.42	30.30
C(7)	29.0	29.2	158.1	H(7)	1.27	1.33	30.41
C(8)	28.9	29.2	158.1	H(8)	1.27	1.37	30.36
C(9)	31.6	30.7	156.5	H(9)	1.27	1.30	30.45
C(10)	20.6	23.1	164.9	H(10)	1.27	1.39	30.33
C(11)	14.0	13.6	175.5	H(11)	0.88	1.02	30.81
a		170.9303		a		24.0232	
b		-0.8962		b		-0.7758	
r^2^		0.9640		r^2^		0.9168	

**Table 5:** Chemical shifts (δ, ppm) in CDCl_3 _and calculated GIAO nuclear magnetic shielding (σ_cal_) for *N,N*-dioctylpyrrolidinium bromide (**3**). The predicted GIAO chemical shifts were computed from the linear equation δ_exp_= a + b·σ_calc_ with a and b determined from the fit the experimental data.

	δ_ exp_	δ_calc_	σ_calc_		δ_exp_	δ_calc_	σ_calc_
C(1)	62.9	61.9	126.0	H(1)	3.85	3.69	27.57
C(2)	21.8	18.6	169.5	H(2)	2.31	1.77	29.81
C(4)	59.4	59.7	128.2	H(4)	3.43	3.32	28.00
C(5)	23.4	24.2	163.9	H(5)	1.70	2.43	29.04
C(6)	26.3	27.5	160.6	H(6)	1.27	1.30	30.49
C(7)	29.0	29.2	158.9	H(7)	1.27	1.26	30.24
C(8)	28.9	30.2	157.8	H(8)	1.27	1.25	30.41
C(9)	31.5	31.1	156.9	H(9)	1.27	1.40	30.40
C(10)	22.5	23.3	164.8	H(10)	1.27	1.18	30.35
C(11)	14.0	12.5	175.6	H(11)	0.88	0.91	30.80
a		187.2433		a		27.4355	
b		-0.9949		b		-0.8611	
r^2^		0.9920		r^2^		0.9049	

The correlation between the experimental chemical shifts and calculated isotropic screening constants are better for ^13^C atoms than for protons. The protons are located on the periphery of the molecule and thus are supposed to be more efficient in intermolecular (solute-solvent) effects than carbons. The differences between the exact values of the calculated and experimental shifts for protons are probably due to the fact that the shifts are calculated for single molecules in gas phase. For this reason the agreement between the experimental and the calculated data for proton is worse than for ^13^C.

## 3. Conclusions

*N,N*-dioctyl-azepanium, -piperidinium, -pyrrolidinium bromides **1-3** have been obtained by reaction of *N,N*-dioctylamine with dibromohexane, dibromopentane and dibromobutane, respectively. The structure of the investigated compounds has been analyzed by FTIR and NMR spectroscopy and B3LYP calculations. Both the FTIR and Raman spectra of **1-3** are consistent with the observed structures in the gas phase. The good correlations between the experimental ^13^C and ^1^H chemical shifts in D_2_O solution and GIAO/B3LYP/6-31G(d,p) calculated isotropic shielding tensors (δ_exp_= a + b·σ_calc_) have confirmed the optimized geometry of **1-3**. 

## 4. Experimental

### 4.1. General

The NMR spectra were measured with a Varian Gemini 300VT spectrometer, operating at 300.07 and 75.4614 MHz for ^1^H and ^13^C, respectively. Typical conditions for the proton spectra were: pulse width 32^o^, acquisition time 5s, FT size 32 K and digital resolution 0.3 Hz per point, and for the carbon spectra pulse width 60^o^, FT size 60 K and digital resolution 0.6 Hz per point, the number of scans varied from 1200 to 10,000 per spectrum. The ^13^C and ^1^H chemical shifts were measured in CDCl_3_ relative to an internal standard of TMS. All proton and carbon-13 resonances were assigned by ^1^H (COSY) and ^13^C (HETCOR). All 2D NMR spectra were recorded at 298 K on a Bruker Avance DRX 600 spectrometer operating at the frequencies 600.315 MHz (^1^H) and 150.963 MHz (^13^C), and equipped with a 5 mm triple-resonance inverse probehead [^1^H/^31^P/BB] with a self-shielded *z* gradient coil (90^o^^1^H pulse width 9.0 μs and ^13^C pulse width 13.3 μs). Infrared spectra were recorded in the KBr pellets using a FT-IR Bruker IFS 66 spectrometer. The Raman spectrum was recorded on a Bruker IFS 66 spectrometer. The ESI (electron spray ionization) mass spectra were recorded on a Waters/Micromass (Manchester, UK) ZQ mass spectrometer equipped with a Harvard Apparatus syringe pump. The sample solutions were prepared in methanol at the concentration of approximately 10^-5^M. The standard ESI – MS mass spectra were recorded at the cone voltage 30V.

### 4.2. Computational Details

The calculations were performed using the Gaussian 03 program package [29] at the B3LYP [30,31] levels of theory with the 6-31G(d,p) basis set [30]. The NMR isotopic shielding constants were calculated using the standard GIAO (Gauge-Independent Atomic Orbital) approach [29,30,31,32] of GAUSSIAN 03 program package [33].

### 4.3. General procedure for the synthesis of N,N-dioctylcycloalkylammonium salts ***1-3***

Dioctylamine (5 g, 0.02 mol) was mixed with the appropriate dibromoalkane (0.02 mol) in the presence of anhydrous sodium carbonate (4.14 g, 0.04mol). The reaction mixture was heated under reflux for 15 h. The solvent was evaporated under reduced pressure and the residue was dried over P_4_O_10_ and then recrystallized from a suitable solvent, as indicated. 

*N,N-dioctylazepanium bromide* (**1**). Prepared from 1,6-dibromohexane (5 g) and recrystallized from acetone/acetonitrile; yield: 65%, m.p. 212-214^o^C. Elemental analysis for C_22_H_46_NBr·H_2_O found (calc.) %C 62.80 (62.53); %H 11.49 (11.45); %N 3.30 (3.31); ES^+^MS *m/z* 325 (C_22_H_46_N); ^1^H-NMR (CDCl_3_): δ 3.70 (4H, t, C(1)H_2_, C(1’)H_2_), 2.01 (4H, m,C(2)H_2_, C(2’)H_2_), 1.79 (4H, m, C(3)H_2_, C(3’)H_2_), 3.45 (4H, t, C(4)H_2_, C(4’)H_2_), 1.71 (20H, m, C(5)H_2_, C(5’)H_2, _C(6)H_2, _C(6’)H_2_, C(7)H_2_, C(7’)H_2_, C(8)H_2_, C(8’)H_2_, C(9)H_2_, C(9’)H_2_), 1.27 (4H, m, C(10)H_2_, C(10’)H_2_), 0.88 (6H, t, C(11)H_3_, C(11’)H_3_); ^13^C-NMR (CDCl_3_): δ 63.1 (C(1), C(1’)), 22.2 (C(2), C(2’)), 27.3 (C(3), C(3’)), 61.3 (C(4), C(4’)), 22.6 (C(5), C(5’)), 26.4 (C(6), C(6’)), 29.1 (C(7), C(7’)), 29.0 (C(8), C(8’)), 31.6 (C(9), C(9’)), 22.6 (C(10), C(10’)), 14.0 (C(11), C(11’)).

*N,N-dioctylpiperidinium bromide* (**2**). From 1,5-dibromopentane (4.76 g, 0.02 mol ). Recrystallized from acetone; yield: 90%, m.p. 144-146^o^C. Elemental analysis for C_21_H_44_NBr found (calc) for %C 64.13 (64.59); %H 12.00 (11.36); %N 3.56 (3.59); ES^+^MS *m/z* 310 (C_21_H_44_N);^ 1^H-NMR (CDCl_3_): δ 3.78 (4H, t, C(1)H_2, _C(1’)H_2_), 1.90 (6H, m, C(2)H_2, _C(2’)H_2_ C(3)H_2_), 3.46 (4H, t, C(4)H_2, _C(4’)H_2_), 1.65 (20H, m, C(5)H_2, _C(5’)H_2, _C(6)H_2, _C(6’)H_2, _C(7)H_2,_ C(7’)H_2,_ C(8)H_2, _C(8’)H_2, _C(9)H_2, _C(9’)H_2_), 1.27 (4H, m, C(10)H_2, _C(10’)H_2_), 0.88 (6H, t, C(11)H_3, _C(11’)H_3_); ^13^C-NMR (CDCl_3_): δ 58.9 (C(1), C(1’)), 20.0 (C(2), C(2’)), 26.4 (C(3)), 58.1 (C(4), C(4’)), 21.7 (C(5), C(5’)), 22.5 (C(6), C(6’)), 29.0 (C(7), C(7’)), 28.9 (C(8), C(8’)), 31.6 (C(9), C(9’)), 20.6 (C(10), C(10’)), 14.0 (C(11), C(11’)).

*N,N-dioctylpyrrolidinium bromide* (**3**). From 1,4-dibromobutane (4.2g, 0.02 mol). Recrystallized from ethyl acetate;yield: 98%, m.p. 120-124^o^C; Elemental analysis for C_20_H_42_NBr found (calc) %C 63.47 (63.81); %H 11.76 (11.24); %N 3.78 (3.72); ES^+^MS *m/z* 296(C_20_H_42_N);^ 1^H-NMR (CDCl_3_): δ 3.85 (4H, t, C(1)H_2,_ C(1’)H_2 _), 2.31 (4H, m, C(2)H_2, _C(2’)H_2_), 3.43 (4H, t, C(4)H_2, _C(4’)H_2_), 1.70 (20H, m, C(5)H_2, _C(5’)H_2, _C(6)H_2,_ C(6’)H_2, _C(7)H_2, _C(7’)H_2, _C(8)H_2, _C(8’)H_2,_ C(9)H_2, _C(9’)H_2_), 1.27 (4H, m, C(10)H_2, _C(10’)H_2_), 0.88 (6H, t, C(11)H_3,_ C(11’)H_3 _); ^13^C-NMR (CDCl_3_): δ 62.9 (C(1), C(1’)), 21.8 (C(2), C(2’)), 59.4 (C(4), C(4’)), 23.4 (C(5), C(5’)), 26.3 (C(6), C(6’)), 29.0 (C(7), C(7’)), 28.9 (C(8), C(8’)), 31.5 (C(9), C(9’)), 22.5 (C(10), C(10’)), 14.0 (C(11), C(11’)).
